# Novel Thienopyrimidine Derivative, RP-010, Induces β-Catenin Fragmentation and Is Efficacious against Prostate Cancer Cells

**DOI:** 10.3390/cancers11050711

**Published:** 2019-05-23

**Authors:** Haneen Amawi, Noor Hussein, Sai H. S. Boddu, Chandrabose Karthikeyan, Frederick E. Williams, Charles R. Ashby, Dayanidhi Raman, Piyush Trivedi, Amit K. Tiwari

**Affiliations:** 1 Department of Pharmacology and Experimental Therapeutics, College of Pharmacy & Pharmaceutical Sciences, University of Toledo, Toledo, OH 43614, USA; haneen.amawi@yu.edu.jo (H.A.); noor.hussein@rockets.utoledo.edu (N.H.); frederick.williams2@utoledo.edu (F.E.W.); 2 Department of Pharmacy Practice, Faculty of Pharmacy, Yarmouk University, P.O. Box 566, Irbid 21163, Jordan; 3 College of Pharmacy and Health Sciences, Ajman University, P.O. Box. 346 Ajman, UAE; s.boddu@ajman.ac.ae; 4 Department of Pharmacy Practice, College of Pharmacy & Pharmaceutical Sciences, University of Toledo, Toledo, OH 43614, USA; 5 School of Pharmaceutical Sciences, Rajiv Gandhi Proudyogiki Vishwavidyalaya, Airport Bypass Road, Gandhi Nagar, Bhopal 462036, India; karthinobel@gmail.com (C.K.); piyushtrivedi304@gmail.com (P.T.); 6 Department of Pharmacy, Indira Gandhi National Tribal University, Amarkantak 484887, India; 7 Department of Pharmaceutical Sciences, College of Pharmacy, St. John’s University, Queens, NY 11432, USA; cnsratdoc@optonline.net; 8 Department of Cancer Biology, College of Medicine, University of Toledo, Toledo, OH 43614 USA; dayanidhi.raman@utoledo.edu; 9 Center of Innovation and Translational Research, Poona College of Pharmacy, Bhartiya Vidyapeeth, Pune 411038, India

**Keywords:** thienopyrimidines, RP-010, prostate cancer, metastasis, Wnt/β-catenin, apoptosis

## Abstract

Thienopyrimidines containing a thiophene ring fused to pyrimidine are reported to have a wide-spectrum of anticancer efficacy in vitro. Here, we report for the first time that thieno[3,2-*d*]pyrimidine-based compounds, also known as the RP series, have efficacy in prostate cancer cells. The compound RP-010 was efficacious against both PC-3 and DU145 prostate cancer (PC) cells (IC_50_ < 1 µM). The cytotoxicity of RP-010 was significantly lower in non-PC, CHO, and CRL-1459 cell lines. RP-010 (0.5, 1, 2, and 4 µM) arrested prostate cancer cells in G2 phase of the cell cycle, and induced mitotic catastrophe and apoptosis in both PC cell lines. Mechanistic studies suggested that RP-010 (1 and 2 µM) affected the wingless-type MMTV (Wnt)/β-catenin signaling pathway, in association with β-catenin fragmentation, while also downregulating important proteins in the pathway, including LRP-6, DVL3, and c-Myc. Interestingly, RP-010 (1 and 2 µM) induced nuclear translocation of the negative feedback proteins, Naked 1 and Naked 2, in the Wnt pathway. In addition, RP-010 (0.5, 1, 2 and 4 µM) significantly decreased the migration of PC cells in vitro. Finally, RP-010 did not produce significant toxic effects in zebrafish at concentrations of up to 6 µM. In conclusion, RP-010 may be an efficacious and relatively nontoxic anticancer compound for prostate cancer. Future mechanistic and in vivo efficacy studies are needed to optimize the hit compound RP-010 for lead optimization and clinical use.

## 1. Introduction

Prostate cancer (PC) is the third leading cause of cancer death in men in the United States, with an estimated 164,690 new cases, along with 29,430 deaths, in 2018 [1]. African-American men usually have the greatest susceptibility to developing PC, compared to other ethnic groups [2]. The majority of men, during the early stage of the disease, are asymptomatic and develop symptoms only during the advanced stages. Treatment of PC is primarily dependent on the age of the patient, disease stage, and aggressiveness of the disease [3]. The majority of localized disease cases can be cured [4]. However, about 20 to 40% of patients previously designated as cured of PC eventually have a recurrence or relapse [5]. Aggressive PC can be treated using androgen deprivation therapy (ADT), often in combination with chemotherapy and/or radiotherapy, but this does not always lead to complete remission [6]. Eventually, PC does not respond to ADT and progresses to highly metastatic castrate-resistant prostate cancer (mCRPC), with a poor prognosis [7,8]. Presently, chemotherapy in mCRPC patients has shown limited benefit, with a survival time of only 16–18 months [8,9]. Consequently, it is essential to develop new treatment strategies, with novel mechanisms of action, that will overcome advanced stages and PC recurrence.

The Wnt/β-catenin signaling pathway is involved in the growth and development of several organs, especially during the embryonic stage [10]. Hyperactivation of this pathway occurs predominantly in colon, ovarian, liver, and prostate cancers [11], and is particularly involved in progression to mCRPC [12]. Therefore, targeting the Wnt signaling pathway, to treat advanced PC, represents a potential novel strategy.

Thienopyrimidines, containing a thiophene ring fused to pyrimidine, are bioisosteres of purines that have been shown to have in vitro anticancer efficacy against certain cancer cell lines [13]. However, there are no previous reports regarding the potential antimetastatic efficacy of any thieno[2,3-*d*]pyrimidine derivatives, nor the mechanism of action, of these compounds [14]. Previously, we reported the synthesis and evaluation of thirteen thieno[2,3-*d*]pyrimidine derivatives (i.e. thieno [2,3-*d*] pyrimidines) [14]. The thienopyrimidine compounds were screened in several cancer cell lines, including colon cancer (HCT116 and HCT115), ovarian cancer (A2780) and brain cancer (LN-229, GBM-10) cell lines. One of the derivatives, compound RP-010, was efficacious against colon cancer cell lines (HCT116, IC_50_ = 0.6 ± 0.3 µM and HCT115, IC_50_ = 0.7 ± 0.2 µM) [14]. Furthermore, RP-010 significantly inhibited cell proliferation in a concentration- and time- dependent manner [14]. To further determine the pharmacological profile of the RP series, we determined their efficacy against the aggressive PC cell lines, PC-3 and DU145. In addition, the potential anticancer mechanism of the most promising compound, RP-010, was also determined, in vitro, in PC cells.

## 2. Results

### 2.1. RP-010 has Efficacy Against PC Cell Lines

**Figure 1:**
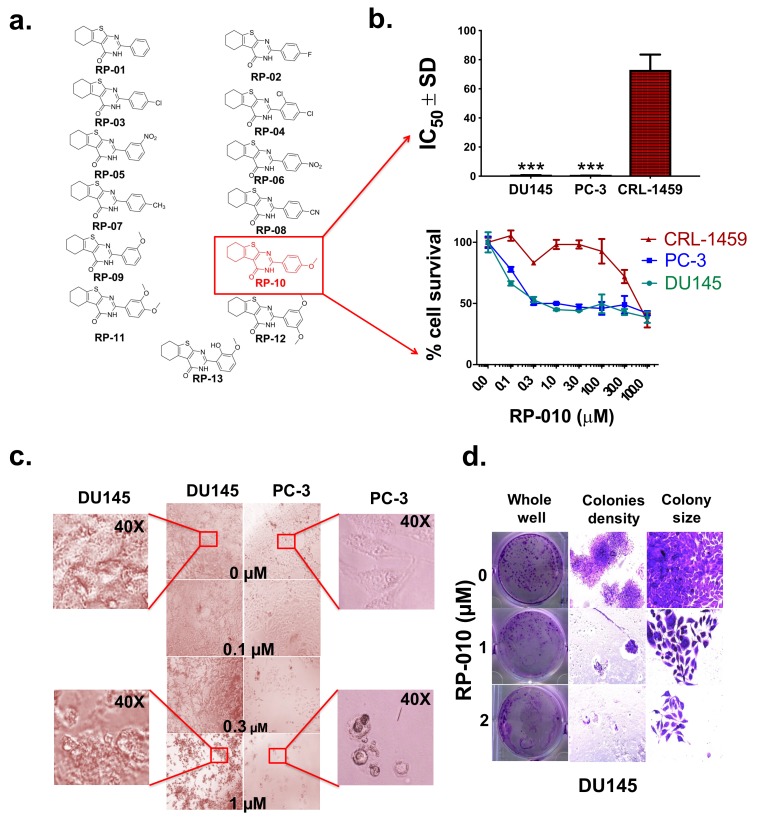
RP-010 cytotoxicity to prostate cancer cells. (**a**) An illustration of the chemical structures of the thirteen RP compounds. (**b**) RP-010 cytotoxicity to prostate cancer cells (DU145 and PC-3), as represented by survival curves (lower panel), and IC_50_ values, compared to non-malignant CRL-1459 cells (upper panel). (**c**) Representative pictures of the morphological changes in cells incubated with RP-010 (0.1, 0.3, or 1 µM), or vehicle, for 72 h. (**d**) Colony formation assay showing the effect of RP-010 or vehicle (1 or 2 µM) on the colony density (10×) and size (20×) of DU145 cells. All results are presented as the means ± SDs of three independent experiments. *** *p* < 0.001.

Among the 13 RP compounds evaluated (Figure 1a and Table 1), the compound RP-010 had the greatest efficacy in the PC cell lines, DU145 (IC_50_ = 0.5 µM) and PC-3 (IC_50_ = 0.3 µM), compared to the non-PC cell lines, CRL-1459 (IC_50_ = 73 µM) and CHO (IC_50_ = 14 µM) (*p* < 0.001 and *p* < 0.01, respectively, Figure 1b and Table 1). Morphological changes in the PC cells, incubated with RP-010 (0.1, 0.3, and 1 µM), compared to the vehicle controls, are shown in Figure 1c. Colony formation assays were used to determine the effect of RP-010 on the number and size of the colonies formed by DU145 and PC-3 PC cells. Colony formation by DU145 cells was significantly decreased by 1 (*p* < 0.01) or 2 µM (*p* < 0.01) RP-010, compared to cells incubated with vehicle (Figure 1d). Furthermore, RP-010 (1 or 2 µM) significantly decreased the size of the colonies formed by DU145 cells, compared to cells incubated with vehicle (Figure 1d). A histogram quantitatively summarizing the results is shown in the supplementary data section (Figure S1). Similarly, the colony formation rate of PC-3 PC cells significantly decreased by 1 (*p* < 0.01) or 2 µM (*p* < 0.01) RP-010, compared to cells incubated with vehicle (Figure S1).

We subsequently determined the efficacy of RP-010 (0.5 or 1 µM) in DU145 PC cells following incubation for 72 h. DU145 cells incubated with vehicle continued to grow and divide over time until they reached ≈ 100% confluence after 72 h (Figure S2). However, DU145 cell growth over time significantly decreased by 1 µM RP-010 (*p* < 0.01) after 12 h of incubation or 0.5 µM of RP10 (*p* < 0.05) after 24 h of incubation compared to cells incubated with vehicle (Figure S2).

### 2.2. RP-010 Blocks the PC Cell Cycle at the G2 Phase

**Figure 2:**
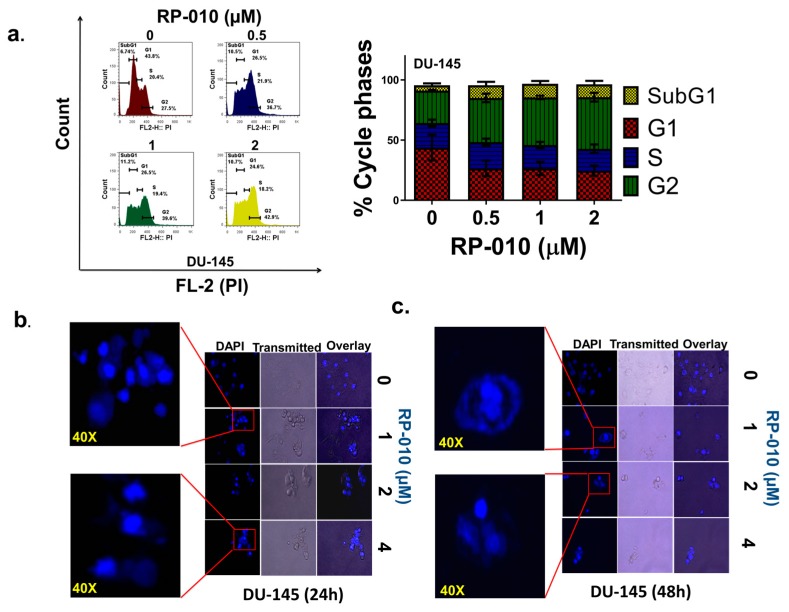
The changes induced by RP-010 on the cell cycle and nuclear events. (**a**) Analysis of RP-010 (0, 0.5, 1, or 2 µM)-induced changes on the cell cycle, using a flow cytometry assay (propidium iodide, “PI,” on the ordinate, and cell count on the abcissa). A graph showing the percent change for each phase, following incubation with RP-010, is shown on the right. In (**b**) and (**c**) the effects of RP-010 (1, 2 or 4 µM) and vehicle on events in the nuclei of DU145 cells, at 24 and 48 h, respectively, are shown. Both chromatin condensation and mitotic catastrophe can be seen.

RP-010 significantly altered the distribution of the DU145 cells in the cell cycle (Figure 2a), producing a significant shift from G1 phase by 0.5 (*p* < 0.05), 1 (*p* < 0.05) or 2 µM (*p* < 0.01) RP-010, compared to cells incubated with vehicle (Figure 2a). The cells then significantly accumulated in the G2 phase, following incubation with 1 (*p* < 0.05) or 2 µM (*p* < 0.05) RP-010, compared to cells incubated with vehicle (Figure 2a). Similarly, there was a significant increase in the percentage of PC-3 cells in the G2 phase, following incubation with 0.5 (*p* < 0.01), 1 (*p* < 0.01) or 2 µM (*p* < 0.0001) of RP-010 (Figure S3). In contrast to DU145 cells, there was a significant decrease in the percentage of PC-3 cells in G1, following incubation with 0.5 (*p* < 0.01), 1 (*p* < 0.01) or 2 µM (*p* < 0.001, Figure S3) of RP-010. Overall, our results indicated that RP-010 arrests PC cells in the G2 phase of the cell cycle.

### 2.3. RP-010 Increases Oxidative Stress in PC Cells

2′,7′-dichlorodihydrofluorescein diacetate (H2DCFDA or DCF) was used to determine the effects of RP-010 (0.5, 1, 2, or 4 µM), or vehicle, on the level of oxidative stress in PC cells (DU145 and PC-3), after 24-h treatment. RP-010 produced a higher fluorescence of DCF in cells incubated with RP-010, compared to cells incubated with vehicle (Figure S4). Moreover, DU145 cells produced significantly higher levels of reactive oxygen species (ROS), following 0.5 µM (*p* < 0.05), 1 µM (*p* < 0.01), 2 µM (*p* < 0.01), or 4 µM (*p* < 0.001) RP-010 treatment, compared to cells treated with vehicle (Figure S4). In PC-3 cells, RP-010 also elevated ROS levels at 1 µM (*p* < 0.05), 2 µM (*p* < 0.05), and 4 µM (*p* < 0.01) compared to vehicle-treated cells (Figure S4).

### 2.4. RP-010 Kills DU145 and PC-3 PC Cells by Mitotic Catastrophe and Apoptosis

The results shown in Figure 2b,c and 5S indicate that RP-010 induced DU145 and PC-3 cell death by two major mechanisms: (1) the formation of giant cells with multi-nuclei (multinucleated giant cells), primarily at 1 µM; and (2) the induction of apoptotic death, predominantly at higher (e.g., 4 µM) concentrations (Figure 2b,c). Analogously, vehicle-treated DU145 cells produced few or no apoptotic cells (Figure 2b,c), while those treated with 1 µM RP-010 for 24 h produced multinucleated cells (Figure 2b). However, the 24 h incubation of DU145 cells with 2 or 4 µM RP-010 significantly increased numbers of highly condensed, fragmented nuclear chromatin, indicative of late-stage apoptosis (Figure 2b). Similarly, treatment of DU145 cells with 1, 2 or 4 µM of RP-010, for 48 h, primarily produced early stage apoptotic nuclear morphology (Figure 2c). The effect of RP-010 on PC-3 cells is shown in Figure S5a,b. Moreover, incubation of PC-3 cells with 1 µM of RP-010 for 24 and 48 h produced a significant increase in multinucleated cells and apoptosis at 2 or 4 μM (Figure S5).

### 2.5. RP-010 Induces Apoptosis by the Pro-Apoptotic Bcl-2 Family Proteins, Inducing Caspase Activation

**Figure 3:**
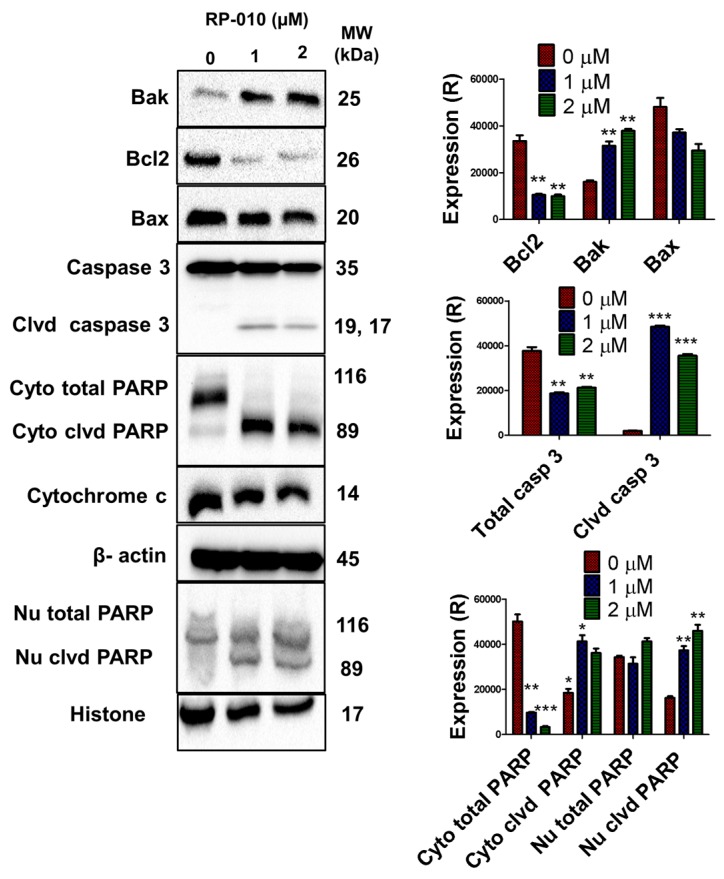
RP-010 activates the intrinsic apoptosis pathway. (Left) The effect of RP-010 (1 or 2 µM, at 24 h incubation) or vehicle on the expression levels of the apoptotis-regulating proteins Bak, Bcl2, caspase-3, cleaved caspase-3, cytosolic (“Cyto”) and nuclear (“Nu”) poly ADP-ribose polymerase (PARP), cytosolic and nuclear cleaved PARP, and cytochrome c, are shown. β-actin and histone were used as reference proteins. (Right) The histograms represent a quantitative summary of the results. (R) = relative. ** *p* < 0.01, *** *p* < 0.001.

We next determined the in vitro effect of 24-h treatment with vehicle or 2 μM RP-010, on expression of the apoptosis-regulating proteins Bak, Bax, Bcl2, caspase 3, and poly ADP-ribose polymerase (PARP), in DU145 cells. The expression level of Bak, in DU145 cells, significantly increased (≥2-fold), following incubation with 1 (*p* < 0.01) or 2 μM (*p* < 0.01) of RP-010, compared to cells incubated with vehicle (Figure 3). In contrast, the incubation of DU145 cells with 1 or 2 μM RP-010 did not significantly alter the expression of the pro-apoptotic protein, Bax (Figure 3). Moreover, the anti-apoptotic protein, Bcl2, was significantly downregulated by 1 (*p* < 0.01) or 2 μM (*p* < 0.01) RP-010, compared to cells incubated with vehicle (Figure 3).

Cleavage of caspase-3, due to the cleavage of inactive caspase-3 (a late-stage event in apoptosis), was significantly increased by incubating DU145 cells with 1 μM (*p* < 0.001) or 2 μM (*p* < 0.001) RP-010 (Figure 3). Subsequently, caspase 3-mediated cleavage of both cytosolic and nuclear PARP, was significantly increased in DU145 cells, following incubation with 1 μM (cytoplasm: *p* < 0.05, nucleus: *p* < 0.01) or 2 μM (cytoplasm: *p* < 0.05, nucleus: *p* < 0.01) RP-010, compared to cells incubated with vehicle (Figure 3). In addition, levels of total cytosolic PARP were significantly reduced by 1 μM (*p* < 0.01) or 2 μM (*p* < 0.001), compared to cells incubated with vehicle (Figure 3). Furthermore, levels of cleaved cytosolic PARP significantly increased by 1 (*p* < 0.05) or 2 μM RP10 (*p* < 0.05), compared to cells incubated with vehicle (Figure 3). Similarly, levels of cleaved nuclear PARP increased following incubation with 1 μM (*p* < 0.01) or 2 μM (*p* < 0.01) RP-010, compared to vehicle-treated cells (Figure 3). However, incubation of DU145 cells with 1 or 2 μM of RP-010 did not significantly alter levels of cytochrome c, compared to cells incubated with vehicle (Figure 3).

### 2.6. RP-010 Inhibits PC Cell Migration

**Figure 4:**
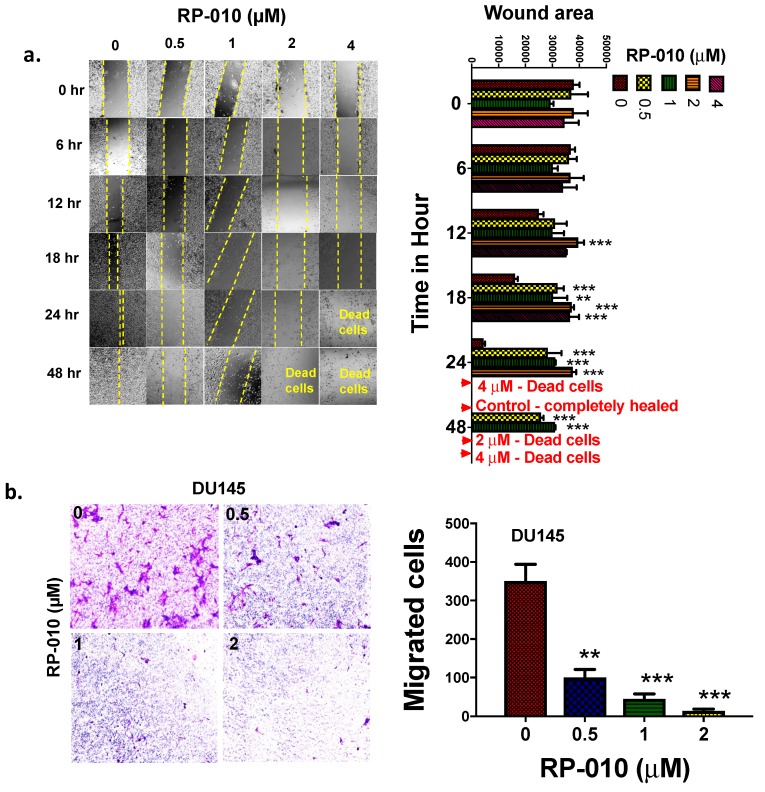
RP-010 significantly reduces cell migration in DU145 cells. (**a**) Results of wound healing assays following incubation with RP-010 (0.5, 1, 2, or 4 µM) or vehicle, with the histogram (right panel) representing a quantitative summary of the results; (**b**) Cell migration following incubation with RP-010 (0.5-, 1- or 2-μM) or vehicle. The histogram (right panel) represents a summary of the results. ** *p* < 0.01, *** *p* < 0.001. The micrograph images were taken at 4× (scale bar: 1/3rd width/box is 1000 μm).

DU145 and PC-3 cells are highly migratory, and thus can be used as an in vitro model to assess one aspect of metastasis [15]. Therefore, we used wound-healing and transwell (a surrogate for invasion) migration assays to determine the effect of RP-010 on DU145 cell migration. In the wound-healing assay, RP-010-treated DU145 cells migrated significantly less than cells incubated with vehicle (Figure 4a). Indeed, there was a significant decrease in DU145 cell wound healing following incubation with RP-010. Results were as follows: 1) at 12 h, (*p* < 0.001 for 2 µM only); 2) at 18 h (*p* < 0.001 for all concentrations); 3) at 24 h (*p* < 0.001) for 0.5, 1 and 2 µM, whereas most of the cells were nonviable at 4 µM (Figure 4a) and 4) at 48 h (*p* <0.001) for 0.5 and 1 µM. The majority of the cells were nonviable (i.e., floating) after incubation with 2 or 4 µM RP-010 (Figure 4a), compared to cells incubated with vehicle. In contrast, vehicle-treated DU145 cells rapidly closed the wounded area, with complete closure detected at 48 h (Figure 4a). In PC-3 cells, the effects of RP-010 on wound closure were similar to those for DU145 cells, with RP-010 treatment significantly decreasing wound healing, as follows: at 12 h (*p* < 0.01 for 0.5 and *p* < 0.001 for 1, 2, and 4 µM); 2) at 18 h (*p* < 0.001 for all concentrations); and 3) at 24 h (*p* < 0.001 for all concentrations). At 48 h, most cells were dead after incubation with 0.5, 1, or 2 µM RP-010 (Figure S6). Similar to DU145 cells, the induced wound in PC-3 monolayers, incubated with vehicle only, closed completely within 48 h (Figure S6).

In the transwell migration assay, the numbers of DU145 cells migrating across the polycarbonate membranes were significantly decreased, following 24 h treatment with 0.5- (*p* < 0.05), 1- (*p* < 0.001), or 2-μM (*p* < 0.001) RP-010, compared to cells incubated with vehicle (Figure 4b). However, PC-3 cell transwell migration was not significantly altered by 0.5-μM (24 h incubation) RP-010, compared to control cells (Figure S6), although their migration was significantly decreased by 24-h treatment with 1- (*p* < 0.05) or 2- (*p* < 0.05) μM RP-010 (Figure S6).

### 2.7. RP-010 Downregulates Wnt/β-Catenin Signaling Pathway Proteins in PC Cells, In Vitro

**Figure 5:**
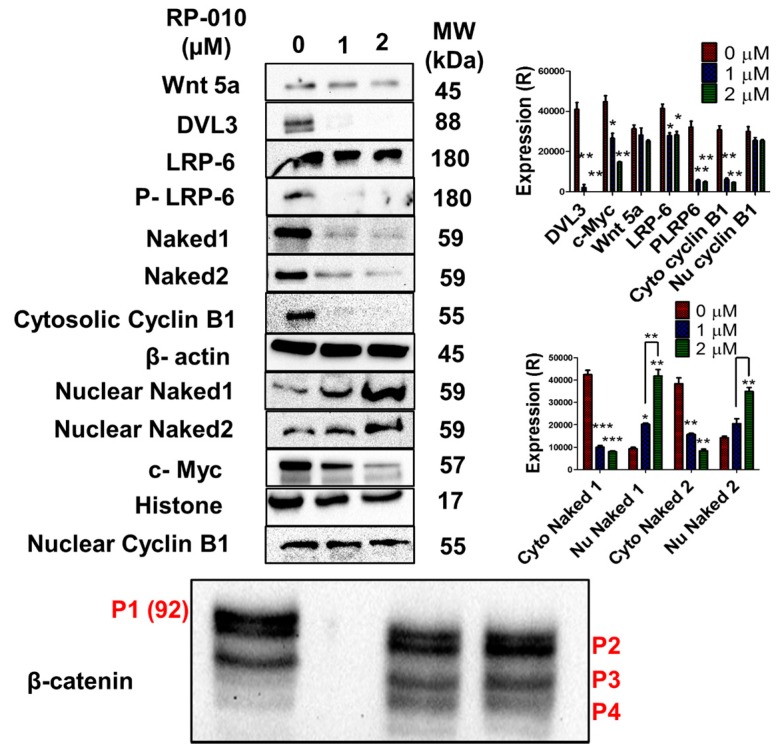
RP-010 induces significant changes in Wnt/ β-catenin signaling proteins. (Left) The effect of RP-010 (1 or 2 µM, after 24 h of incubation), or vehicle, on the expression levels of the following proteins: Wnt 5a, DVL3, LRP-6, P-LRP-6, cytosolic and nuclear Naked1 and 2, cytosolic and nuclear cyclin B, c-Myc, and β-catenin (bottom panel). β-actin and histone were used as reference proteins. The histograms (right panels) represent quantitative summaries of the results. (R) = relative. * *p* < 0.05, ** *p* < 0.01, *** *p* < 0.001.

We next determined the effect of RP-010 (24-h incubation with 0, 1 or 2 μM) on the expression levels of the following nuclear and cytosolic proteins of the Wnt/β-catenin signaling pathway: (1) Wnt 5a; (2) DVL3; (3) LRP-6; (4) P-LRP-6; (5) Naked 1; (6) Naked 2, (7) cyclin B1; (8) c-Myc; (9) nuclear cyclin B1 and (10) β-catenin, in DU145 PC cells. The levels of the pro-growth protein DVL3, in DU145 cells, significantly decreased by 1 (*p* < 0.01) or 2 μM (*p* < 0.01) RP-010, compared to cells incubated with vehicle (Figure 5).

In addition, levels of the Wnt ligand receptor LRP-6, in DU145 cells, were significantly decreased by 1 μM RP-010 (*p* < 0.05) or 2- (*p* < 0.05) (Figure 5). Similarly, levels of phosphorylated LRP-6 (i.e., P-LRP-6), were significantly decreased in DU145 cells by 1 or 2 μM RP-010 (*p* < 0.01 for both concentrations of RP-010) (Figure 5).

The incubation of DU145 cells with RP-010 significantly decreased the levels of cytosolic Naked 1 (*p* < 0.001 for 1 or 2 μM) and Naked 2 (*p* < 0.01 for 1 or 2 μM) proteins, compared to cells incubated with vehicle (Figure 5). In contrast, incubation of these cells with RP-010 significantly increased nuclear levels of Naked 1 (*p* < 0.05 for 1 μM and *p* < 0.01 for 2 μM) and Naked 2 (*p* < 0.05 for 1 μM and *p* < 0.01 for 2 μM), compared to cells incubated with vehicle (Figure 5).

The incubation of DU145 cells with 1 or 2 μM RP-010 did not significantly alter the levels of the epithelial marker E-cadherin, compared to cells incubated with vehicle (Figure S8). Moreover, cytosolic levels of cyclin B1 decreased significantly by treatment with 1- or 2-μM RP-010 (*p* < 0.01 for both concentrations), whereas its nuclear levels were not significantly altered, compared to cells incubated with vehicle (Figure 5). In addition, levels of the nuclear c-Myc oncoprotein, in DU145 cells, significantly decreased by 1 (*p* < 0.05) or 2 μM (*p* < 0.01) RP-010, compared to cells incubated with vehicle (Figure 5).

In DU145 cells, RP-010 (1 or 2 µM) produced significant fragmentation of the high molecular weight, full-length β-catenin protein, p92 (or p1), into several lower molecular weight fragments (≈70 kDa) (Figure 5 and Figure S7). There was a significant decrease in the levels of total active β-catenin in DU145 cells incubated with 1- (*p* < 0.01) or 2- (*p* < 0.001) μM RP-010 (Figure 5 and Figure S7). This RP-010-induced cleavage of total active β-catenin increased the accumulation of its lower molecular weight fragment, compared to cells incubated with vehicle, a form that is primarily inactive, including its fragments P3 (*p* < 0.01 for 1 or 2 µM), and P4 (*p* < 0.05 for 1 or 2 µM) (Figure 5 and Figure S7). Interestingly, no significant change in the levels of Wnt 5a was detected, following incubation with RP-010, compared to cells incubated with vehicle (Figure 5).

### 2.8. Effect of RP-010 in an In Vivo Zebrafish Model of Toxicity

**Figure 6:**
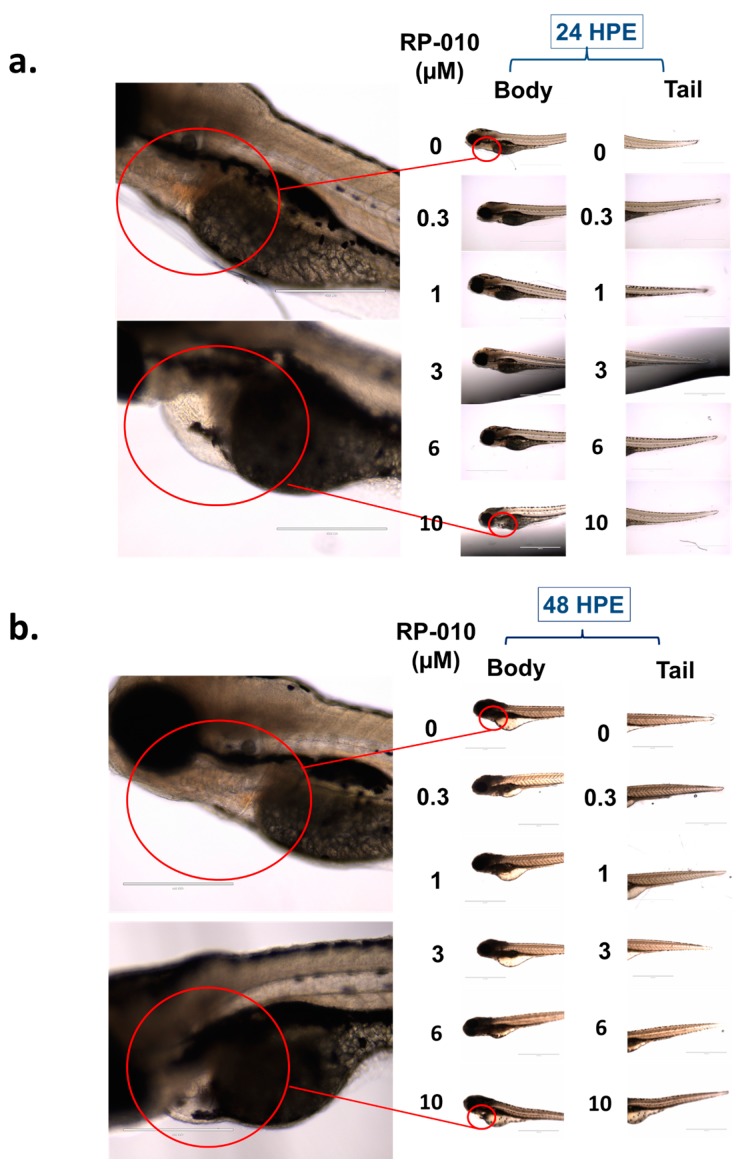
RP-010 toxicity in an in vivo zebrafish model. The effect of RP-010 (0, 0.3, 1, 3, 6, or 10 µM), or vehicle treatment, on the bodies (left) and tails (right) of zebrafish, after (**a**) 24- or (**b**) 48-h drug exposure. Scale bar: Images were taken at 4× (scale bar: 1000 μm) and zoomed to 10× (scale bar: 400 μm).

After establishing the anticancer activity of RP-010, we used an in vivo zebrafish model to assess its potential toxic effects. The exposure (24 and 48 h) of zebrafish to 0, 0.3, 1, 3, 6, or 10 µM RP-010 did not significantly increase mortality, compared to vehicle (Figure 6). None of the concentrations of RP-010 significantly altered the body or tail shapes or their lengths compared to vehicle (Figure 6a,b). Furthermore, no malformations were detected following exposure to RP-010 (Figure 6a,b). However, at 10 µM, RP-010 produced significant pericardial edema (*p* < 0.05) after 24- or 48-h exposure (Figure 6a,b), compared to exposure to the vehicle. Furthermore, the zebrafish developed a significant loss in dorsoventral balance following 24 or 48 h of exposure to 10 µM of RP-010, compared to vehicle (Figure 6a,b). A significant increase in pericardial edema and irregularities in heart rate were observed (*p* < 0.05), following 24 or 48 h of exposure to 10 μM of RP-010 compared to vehicle (Figure 6a,b). The effect of RP-010 on zebrafish heart rate is shown in Figure S9. Overall, our results indicated that RP-010 was well tolerated by zebrafish at concentrations of up to 6 µM.

## 3. Discussion

In this study, we assessed the efficacy and mechanism of action of the novel thienopyrimidine derivative, RP-010, in PC cells. The thieno[2,3-*d*]pyrimidines were synthesized to structurally resemble natural purines, and the synthetic 4-anilinoquinazoline core in FDA-approved anticancer drugs such as gefitinib, erlotinib [16], and tandutinib [17]. The in vitro anticancer efficacy of these thienopyrimidine derivatives has been previously reported [18]. The IC_50_ values in hepatocellular carcinoma cells (HEPG2) for the thienopyrimidine and triazolothienopyrimidine derivatives were 2-3 µM [18]. In the breast cancer line, MCF-7, the IC_50_ was ≈ 4 µM for the thienopyrimidines derivatives, and 15 μM for the triazolothienopyrimidine derivatives [19]. Furthermore, thieno[2,3-*d*] pyrimidine derivatives containing a thiosemicarbazide moiety (compounds 5a-d) had IC_50_ values from 5.3–138 μM in PC-3 cells [20]. In contrast, our synthesized thieno[2,3-*d*]pyrimidines compounds had anticancer efficacy in PC-3 and DU145 PC cells, and the compound RP-010 had the highest potency of all of the 13 derivatives (IC_50_ < 1 μM). Previously, it has been reported that the anticancer efficacy of the thienopyrimidine compounds may be due to inhibition of cyclin kinase, inhibition of thymidylate synthase, and antagonism of gonadotropin-releasing hormone (GnRH) receptor [19,21,22]. The overexpression of the epidermal growth factor receptor (EGFR) and the protein, v-erb-b2 erythroblastic leukemia viral oncogene homolog 2 (ErbB2), have been reported to be involved in cancer progression and development, including prostate cancer [23]. Thienopyrimidines with two different cores (thieno [3, 2-*d*] pyrimidine (core A) and thieno [2,3-*d*] pyrimidine (core B)) were reported and compounds that were derivatives of core A inhibited EGFR and ErbB2, with IC_50_ values of 1–68 nM and 30–705 nM, respectively [24].

The incubation of DU145 and PC-3 PC cells with 1-, 2-, or 4-µM RP-010 induced the formation of single cells with multiple nuclei, especially at low concentrations. This morphological change in different cancer cells has been previously reported [25]. This phenomenon has been designated “mitotic catastrophe” or polyploidy, a unique mechanism of death that occurs during mitosis [26]. Cells that undergo mitotic catastrophe typically have a reduced proliferative capacity and become flattened and enlarged [27]. Previously, it has been reported that reversine, an inhibitor of aurora kinase that also induces apoptosis and mitotic catastrophe, has efficacy against certain human cancers [28]. Of note, reversine induces the formation of multinucleated, giant cells in human prostate, breast, and lung cancer cell lines [29,30]. Interestingly, doxorubicin, an FDA-approved anticancer drug, induces cell death by apoptosis, at high concentrations, and mitotic catastrophe, at lower concentrations (50 ng/mL), in Huh-7 hepatoma cells that exit mitosis without cell division, forming a single giant cell with multiple nuclei and decondensed chromatin [31]. Moscatilin, a constituent of orchids, also produces the aforementioned changes in certain cancer cell lines in vitro [32]. This type of cell death could result from an alteration in the regulators of mitosis, including cell cycle-specific kinases (e.g., the cyclin B1-dependent kinase Cdk1, polo-like kinases, and aurora kinases), survivin, p53, caspases, and Bcl-2 family proteins [29]. Currently, the effect of RP-010 on these proteins remains to be elucidated. Since Bcl2 proteins are important regulators of the intrinsic pathway of apoptosis [33,34], their activation results in activation of caspases, which cleave many cellular proteins, including PARP [35]. In addition to multinuclear formation, RP-010 also induced apoptotic chromatin condensation, in both PC cell lines, particularly at higher concentrations.

During the activation of the canonical Wnt/β-catenin signaling pathway [36], following Wnt ligand binding to its membrane receptors (Frizzled) and the low-density lipoprotein receptor-related protein 6 co-receptors (LRP-6), dishevelled (Dvl) and axin are recruited to a large protein complex, resulting in inhibition of β-catenin phosphorylation and its degradation [37]. Subsequently, activated β-catenin is translocated to the nucleus [38], where it interacts with specific transcriptional cofactors, (T-cell factor TCF) and lymphoid enhancer factor (LEF), inducing the expression of Wnt-responsive proteins such as c-Myc, MMP-7, cyclins, as well as other proteins [39]. This overactivation and accumulation of β-catenin in the nucleus induces the progression of colon, ovarian, liver, and prostate cancers, among others [10,40,41]. Wnt/β-catenin signaling also plays a critical role in cancer cell metastasis and invasiveness, by facilitating the epithelial-to-mesenchymal transition [42]. In prostate cancer, β-catenin elicits progression and growth in a transgenic mouse model, even in the absence of Wnt signaling [43]. β-catenin can produce highly invasive prostate cancer in several transgenic mouse models (e.g. with SV40 large T-antigen, loss of PTEN, mutated K-ras) [42]. Furthermore, β-catenin nuclear localization was detected in 24% of metastatic samples from patients with metastatic castrate-resistant prostate cancer (mCRPC) [44]. β-catenin expression was significantly increased in 38% of patients with mCRPC, compared to 23% of patients with localized, non-metastatic prostate cancer [45]. In 11 of 27 mCRPC patients with bone metastasis, nuclear localization of β-catenin was detected [46]. Therefore, the inhibition of this pathway or the key regulator proteins of this pathway, may represent a novel mechanism to inhibit prostate cancer progression and benefit in mCRPC stage [47]. β-catenin has been reported to be biotransformed in HeLa cells, resulting in multiple lower molecular weight products, with little or no transcriptional efficacy [48]. Previously, we reported β-catenin fragmentation in HCT116 colon cancer cells, following incubation with 15k, a novel derivative of silybin, a constituent of milk thistle seeds [49,50]. In this study, the incubation of prostate cancer cells with RP-010 induced degradation of nuclear β-catenin into smaller fragments, potentially with different or lower transcriptional activity, in prostate cancer cells. Thus, RP-010-induced fragmentation of β-catenin is a major cellular event, which may account for its antiproliferative and antimetastatic efficacy.

Additional proteins in the Wnt/β-catenin signaling pathway also play essential roles in the activation of this pathway. For example, LRP-6 receptors are required to signal the early events produced by Wnt binding in the cytosol [37]. The intracellular domain of LRP6 is phosphorylated at specific serine and threonine residues by GSK3 and casein kinase (CK), upon activation of Wnt signaling [51]. The hyaluronan-binding protein CD44 also activates LRP-6 phosphorylation, upregulating other proteins in the Wnt signaling pathway, and inhibition of LRP-6 phosphorylation downregulates Wnt/β-catenin signaling [52]. For example, triptolide, a diterpene epoxide derived from a natural product, inhibits LRP-6 phosphorylation, and pancreatic cancer proliferation, in a mouse model (KPC (Kras^G12D^, P53^R172H^, PDX^Cre^) of pancreatic-derived tumors [53]. In the study described herein, RP-010 significantly downregulated LRP-6 expression. RP-010 produced a significantly greater inhibition of the phosphorylation of LRP-6 instead of downregulating the total protein level, thereby contributing to its multi-target mechanism of action. Disheveled protein 3 (Dvl3) also plays a role in β-catenin activation and stabilization, by preventing phosphorylation of β-catenin by GSK3B [37]. Upregulated Dvl3 was also recently investigated as a biomarker for the recurrence of prostate cancer [54], while its downregulation sensitizes prostate and breast cancer cells to insulin-like growth factor 1 (IGF1) [55]. Therefore, the inhibition of Dvl3 protein can decrease β-catenin activation, and its subsequent nuclear translocation [56]. RP-010, in vitro, significantly downregulated cytosolic levels of Dvl3 protein, thus producing additional inhibition of the Wnt/β-catenin signaling pathway.

The proteins Naked cuticle homologs 1 and 2 (Naked 1 and Naked 2) are important downregulators of the Wnt signaling pathway [36], and inhibition of their activity increases Wnt signaling overactivation and subsequent cancer cell growth and proliferation [57]. Analogously, epigenetic silencing of *Naked 2* gene by methylation of its promoter region induced breast cancer growth and proliferation in 7 distinct breast cancer cell lines [58]. Naked 2 also reverses the effects of Wnt/β-catenin in both zebrafish and HEK293 embryonic kidney cells [59]. Naked 1 forms a Tcf/β-catenin/Naked1 heterotrimeric complex, and negatively regulates Wnt signaling in human colorectal cancer cells [36]. In zebrafish embryos, Naked 1 binds to β-catenin and inhibits its nuclear translocation [60]. Likewise, *Naked 2* promoter methylation-induced silencing facilitates gastric cancer proliferation, metastasis and migration, both in vitro (gastric cancer cell lines) and in vivo (gastric cancer xenograft mouse model). However, restoration of Naked 2 efficacy and expression inhibited cancer growth, induced G2/M cycle arrest, inhibited cell migration, and restored the anticancer efficacy of docetaxel [61]. Moreover, RP-010 (1 or 2 µM) significantly induced the translocation of Naked 1 and Naked 2 proteins from the cytosol to the nucleus, as indicated by significantly decreased Naked 1 and Naked 2 levels in the cytosol with concomitantly increased nuclear levels. This in vitro finding is novel and may, in part, contribute to RP-010’s anticancer efficacy. Further experiments are required to delineate the effects of RP-010 on the nuclear functions and transcriptional activities of Naked 1 and Naked 2.

Currently, the exact role of cell cycle regulatory protein, cyclin B1 in cancer cell growth development, progression and metastasis is not completely understood [62,63]. It has been shown that beneficial effects of imiquimod results from inhibition of cyclin B1-induced G2 cell cycle arrest and apoptosis in PC cells, in vitro and in vivo [64]. In contrast, upregulation of cyclin B1 reverses metastasis in colorectal cancer cells [65]. Several anticancer drugs (e.g., flavopiridol and palbociclib) is known to significantly inhibit the expression and activity of cellular cyclins, such as cyclin B1 [66]. We found that RP-010 (1 or 2 µM) significantly decreased cytosolic cyclin B levels, without significantly altering its nuclear levels, resulting in chemosensitization of DU145 PC cells, and inhibition of proliferation. The *myc* gene (*c-Myc*) codes for the protein, c-Myc, which induces significant changes in Wnt signaling by inhibiting GSK3β activity, i.e., phosphorylation and inactivation of β-catenin [67]. To that end, c-Myc is upregulated by nuclear translocation of β-catenin [68]. RP-010 (1 or 2 µM) significantly decreased the expression levels of c-Myc, to a greater magnitude in DU145 cells, compared to PC-3 cells, suggesting decreased nuclear translocation of β-catenin. However, this remains to be further validated.

The epithelial mesenchymal transition (EMT) is involved in the development, progression, metastasis, and drug-resistance of cancer [69]. Cancer cells with the EMT phenotype can migrate from the extracellular matrix to reach distant tissues [70]. The Wnt/β-catenin-signaling cascade is predominantly involved in EMT progression [71], with the latter characterized by loss of E-cadherin protein and upregulation of N-cadherin [72,73]. However, RP-010 (1 and 2 µM) did not significantly alter the expression levels of either E-cadherin or N-cadherin. These results suggest that RP-010 does not significantly affect EMT, and that the anticancer efficacy of RP-010 may mainly occur through the induction of apoptosis and inhibition of Wnt/β-catenin signaling.

## 4. Materials and Methods

### 4.1. Reagents

Primary and secondary antibodies were purchased from Cell Signaling Technology (Danvers, MA, USA). Propidium iodide (PI) dye was purchased from Life Technologies (Eugene, Oregon, USA). Dulbecco’s modified Eagle’s medium (DMEM) was purchased from GE Healthcare Life Sciences, HyClone Laboratories (Logan, UT, USA). The 0.25% trypsin + 2.2 mM ethylenediaminetraacetic acid (EDTA) and phosphate-buffered saline (PBS) were purchased from Mediatech, Inc. (Corning subsidiaries, Manassas, VA, USA). Crystal violet dye powder was purchased from Sigma-Aldrich (St. Louis, MO, USA). Corning^®^ transwell inserts (24 mm Transwell with 8.0-μm pore polycarbonate membrane inserts, TC-treated, w/lid, sterile, 24/cs) were purchased from Sigma-Aldrich (St. Louis, MO, USA). The compound 3-(4,5dimethylthiazol-2-yl)- 2,5-diphenyltetrazolium bromide (MTT) was purchased from Calbiochem EMD Millipore (Billerica, MA, USA). Clarity™ and Clarity Max™ Western ECL blotting substrates were purchased from Bio-Rad Laboratories (Hercules, CA, USA).

### 4.2. Cell Lines and Culture

The prostate cancer cell lines, DU145 and PC-3, as well as non-prostate CRL-1459 (human normal colon epithelial cell line) and CHO (Chinese hamster ovarian) cell lines were a kind gift from the late Dr. Gary Kruh. All the cells were cultured as adherent monolayers in culture flasks, and were used to determine the anti-cancer efficacy of RP-010. DMEM supplemented with 4.5 g of glucose, 10% fetal bovine serum (FBS) and 1% penicillin/streptomycin, was used to augment cell growth. Finally, the cells were incubated and grown in a humidified incubator containing 5% CO_2_ at 37 °C [50]. All cells were authenticated to be free of fungi and mycoplasma. Cells were obtained from frozen stocks and cell passaging (up to P4) was performed at 80% cell confluency, using PBS and trypsin + 2.2-mM EDTA.

### 4.3. Cell Cytotoxicity Assays

#### 4.3.1. MTT Assay

MTT assays were used to determine the cytotoxicity of the RP compounds, to PC cell lines, as previously described [74]. The RP compounds (1–13) were evaluated in each cell line at concentrations of 0, 0.1, 0.3, 1, 3, 10, 30 and 100 µM. A comparison was made between the cytotoxicity of RP-010 in PC and the non-PC i.e. CHO and CRL-1459 cell lines.

#### 4.3.2. Colony Formation Assay

This assay was performed as previously described [50]. The DU145 and PC-3 cells were incubated with RP-010 (1 or 2 µM) for 24 h. After 12 h of incubation, the medium was discarded and cells were trypsinized with 0.25% trypsin, 2.21-mM EDTA, and then harvested, counted, and reseeded in 6-well plates at a low density (500 cell/well). The cells were allowed to form colonies for 10–14 days at 37 °C, with the medium being changed every other day. Subsequently, methanol was used to fix the colonies formed in each plate, followed by staining the colonies with 0.1% crystal violet dye for 30 min. Finally, colonies were viewed and counted under an EVOS microscope (Themo Fisher Scientific, Wayne, MI, USA). The colony formation rate equation was applied for each compound: Colony formation rate = the number of colonies/numbers of seeded cells × 100%.

#### 4.3.3. Time-Dependent Studies with RP-010

The cytotoxicity of RP-010, over time, was determined using DU145 cells. The cells were seeded and incubated as previously described [49]. The DU145 cells were incubated with vehicle or RP-010 for 72 h. The pictures were taken over time, at a snapshot interval of 15 min, from the same spot of the flask using the CytoSMART™ Lux 10X system (LONZA biosciences Walkersville, MD, USA) for live cell imaging.

### 4.4. Cell Cycle Analysis

The effect of RP-010 on the cell cycle phases and function was determined using cell cycle analysis with PI staining, followed by flow cytometry [74,75]. The cells were incubated with either vehicle or RP-010 (0.5, 1 or 2 µM) for 24 h. After incubation overnight, the cells were collected, washed and resuspended as an ice-cold PBS suspension. The DNA was stained with PI for at least 15 min on ice. A FACS Calibur flow cytometer from BD Biosciences (San Jose, CA, USA) was used to determine the distribution of the cells following incubation with vehicle or RP-010. Finally, the data were viewed and analysed using FlowJo v10.2 software from FlowJo LLC (Ashland, OR, USA).

### 4.5. Detection of Oxidative Stress in PC Cells

The compound 2′,7′-dichlorofluorescin (H2DCFDA) was used to detect changes in the oxidative stress status, as previously described [14,50] in PC-3 and DU145. After 24 h of incubation with 0, 0.5, 1, and 4 µM of RP-010, the cells were incubated with H2DCFDA for 30 min at 37 °C. The cells were then washed 3 times with 1× PBS for 5 min each. The level of reactive oxygen species was then measured, based on the fluorescence level of oxidized H2DCFDA dye (excitation at 485 nm and emission at 535 nm), using an EVOS digital fluorescent microscope at 40×.

### 4.6. DAPI Staining

The nuclear changes in DU145 and PC-3 cells lines produced by RP-010 (1, 2 and 4 µM) incubation at 24 or 48 h were determined using DAPI staining. The cells were seeded on culture cover glasses in 6-well plates, and allowed to grow overnight. The cells were then incubated with RP-010, for an additional 24 h, at 37 °C. The incubated cells were fixed with 4% paraformaldehyde, followed by permeabilization with 1× PBS/0.3% Triton X-100 for 25 min. Finally, DAPI stain was added, and cells were photographed using the EVOS microscope.

### 4.7. Migration Assays

#### 4.7.1. Wound Healing Assays

This assay was conducted using DU145 and PC-3 cells as previously described [49,50]. Upon confluence, 200 μL sterile tips were used to create a wound by gently scratching the complete cell monolayer. Sterile PBS was used to wash the cells several times to remove any floating cells. Different concentrations of the test compound were prepared in the culture media and added immediately after wound formation. The closure of the wound was observed by taking pictures at different time points by the EVOS microscope (Thermo Fisher Scientific, Wayne, MI, USA). Finally, the area of the wounds, at different time points, was calculated using Image J software (NIH, Bethesda, MD, USA).

#### 4.7.2. Transwell Migration Assays

The assay was done as previously described [50]. The effect of RP-010, (0.5, 1 or 2 µM) on the migration of PC cells was tested using 24 trans-well inserts with an 8-μM pore size. The addition of the insert to the well forms two chambers (upper and lower), where the upper chamber was used to grow the cells on the porous membrane. The lower chamber was filled with 600-μL cell-free DMEM medium. 200-μL of the cell suspension was added to each insert, and cells allowed to attach for 1 h. The test compound was added in different concentrations and incubated with the cells for 24 h. Cells that did not migrate were removed from the upper chamber by a cotton swab. Finally, the remaining migrated cells were fixed by methanol, and stained with 0.1% crystal violet dye and counted with an EVOS microscope. The number of migrated cells was counted for the cultures incubated with vehicle or RP-010.

### 4.8. Subcellular Fractionation and Western Analysis

DU145 cells were incubated with RP-010 ((1 or 2 µM) for 24 h. Thereafter, the cells were subjected to a two-step lysis process to extract and separate the cytosolic and the nuclear proteins, as previously described [50]. Next, protein separation for both cytosolic and nuclear proteins was achieved by electrophoresing the samples through acrylamide SDS-PAGE gels, followed by transfer to a PVDF membrane. The PVDF membrane was then incubated overnight with the following primary antibodies: rabbit α-tubulin, 1:4000 dilution, rabbit BAX (1:1000), rabbit BAK (1:1000), rabbit cytochrome c (1:1000), rabbit PARP (1:5000), rabbit β-Catenin (1:4000), rabbit DVL3 (1:4000), rabbit LRP-6 (1:1000), rabbit P-LRP-6 (1:1000), rabbit c-Myc (1: 4000), rabbit cyclin B1 (1:2000), rabbit Wnt 5a (1:1000), rabbit naked 1 and naked 2 (1:1000), mouse E-cadherin (1:4000), mouse N-cadherin (1:4000), mouse β-actin (1:5000) and rabbit histone (1:3000). Finally, protein expression levels were determined as described previously [76]. In the Figure 3, Figure 5 and Figure S8, the protein blots above β-actin were cytosolic lysates, while all the proteins below β-actin and above histones (loading controls) were nuclear lysates.

### 4.9. Evaluation of RP-010 Toxicity, in Zebrafish, In Vivo

The zebrafish toxicity studies were conducted as previously described [49]. Five days-post fertilization larvae (5 fish/well) were exposed to vehicle or RP-010 (0, 0.3, 1, 3, 6, and 10 µM). Subsequently, the zebrafish were observed visually, and pictures were taken at 24- and 48-hour post exposure (hpe). The zebrafish were observed for mortality, changes in swimming position, cardiac toxicities (e.g., changes in heart rate and cardiac swelling or oedema), and any morphological changes and malformations of the body and tail. The zebrafish studies were approved by the University of Toledo Institutional Animal Care and Use Committee (#105414, approved on 10/2018).

### 4.10. Statistical Analysis

The data from the wound healing, and cell cycle were analyzed using a two-way analysis of variance (ANOVA), and post-hoc analysis was done using Bonferroni’s multiple comparisons. The data from the colony formation, Western blots, and transwell migration assays were analyzed using a one-way ANOVA, and post hoc analysis was done using Tukey’s multiple comparison tests. All of the experiments were repeated in triplicate. The results were expressed as the mean ± the standard deviation (SD). The a priori significance level was *p* < 0.05.

## 5. Conclusions

RP-010 induced the death of DU145 and PC-3 cells by mitotic catastrophe and induction of apoptosis. RP-010 induced apoptosis by (1) upregulating Bak levels; (2) downregulating Bcl2 expression levels; and (3) cleaving inactive caspase 3 to produce active caspase 3. Furthermore, RP-010 inhibited Wnt/β-catenin pathway signaling, as evidenced by β-catenin fragmentation, while downregulating important proteins in the pathway, including LRP-6, DVL3, and c-Myc. Finally, RP-010 reversed the migration of prostate cells in a time- and concentration-dependent manner, and produced no overt toxicity in an in vivo zebrafish model. If our findings can be extrapolated to humans with PC, RP-010 may be an efficacious compound for the treatment of prostate cancer in advanced stages. Future mechanistic and in vivo efficacy studies are needed to optimize the hit compound RP-010 for clinical use.

## Figures and Tables

**Table 1:** Cytotoxicity data for the RP-010 series compounds (RP-01–RP-013) on prostate cancer (PC) vs. non-PC cell lines.

RP Series	IC_50_ ± SD (µM)			
	Prostate Cancer Cells		Non-Prostate Cells	
	PC-3	DU145	CRL-1459	CHO
RP1	>100	>100	>100	>100
RP2	>100	>100	>100	>100
RP3	>100	>100	>100	>100
RP4	73.4 ± 3.1	>100	>100	>100
RP5	>100	>100	>100	>100
RP6	>100	>100	>100	>100
RP7	>100	>100	>100	>100
RP8	6.4 ± 0.5	>100	>100	>100
RP9	2.8 ± 1.1	>100	>100	>100
RP10	0.3 ± 0.1	0.5 ± 0.2	73.0 ± 5.3	14.0 ± 1.3
RP11	6.6 ± 0.7	2.7 ± 0.5	>100	>100
RP12	6.9 ± 1.0	>100	>100	>100
RP13	>100	>100	>100	>100

MTT assays were used to determine cell survival. IC_50_ values represent as means ± SDs of three independent experiments. Anticancer efficacies of the 13 RP series compounds were screened in DU145 and PC-3 PC cells and compared with the non-PC cells CRL-1459 (normal colon epithelial cell line) and CHO (Chinese hamster ovarian cell line).

## Supplementary Materials

The following are available online at https://www.mdpi.com/2072-6694/11/5/711/s1, Figure S1: The effect of RP-010 on colony formation rate of prostate cancer cells, Figure S2: The effect of RP-010 on cell confluence and viability overtime in DU145 cells, Figure S3: RP-010-induced alterations in the cell cycle in PC-3 cells, Figure S4: The effect of RP-010 on oxidative stress in DU145 and PC-3 cells, Figure S5: The effect of RP-010 on nuclear events in PC-3 cells, Figure S6: RP-010 significantly reduces cell migration in PC-3 cells, Figure S7: RP-010 induces significant changes in Wnt/β-catenin signaling, Figure S8: The effect of RP-010 (1 or 2 µM) or vehicle on the expression of E-cadherin, N-cadherin, α-tubulin, and HSP90, Figure S9: The effect on the heart rate of zebra fish exposed for upto 48 h with (0.3, 1, 3, 6 or 10 µM) or without RP-010.
