# Supplementary material for: Novel Thienopyrimidine Derivative, RP-010, Induces β-Catenin Fragmentation and Is Efficacious against Prostate Cancer Cells
Source: Cancers (Basel). 2019 May 23;11(5):711. doi: 10.3390/cancers11050711 (PMC6563099; doi:10.3390/cancers11050711)
Supplement: Supplementary file 1 [file cancers-11-00711-s001.pdf]

Supplementary Materials: Novel Thienopyrimidine Derivative, RP-010, Induces β-Catenin Fragmentation and is Effecatious Against Prostate Cancer Cells

Haneen Amawi, Noor Hussein, Sai H. S. Boddu, Chandrabose Karthikeyan, Frederick E. Williams, Charles R. Ashby Jr., Dayanidhi Raman, Piyush Trivedi and Amit K. Tiwari

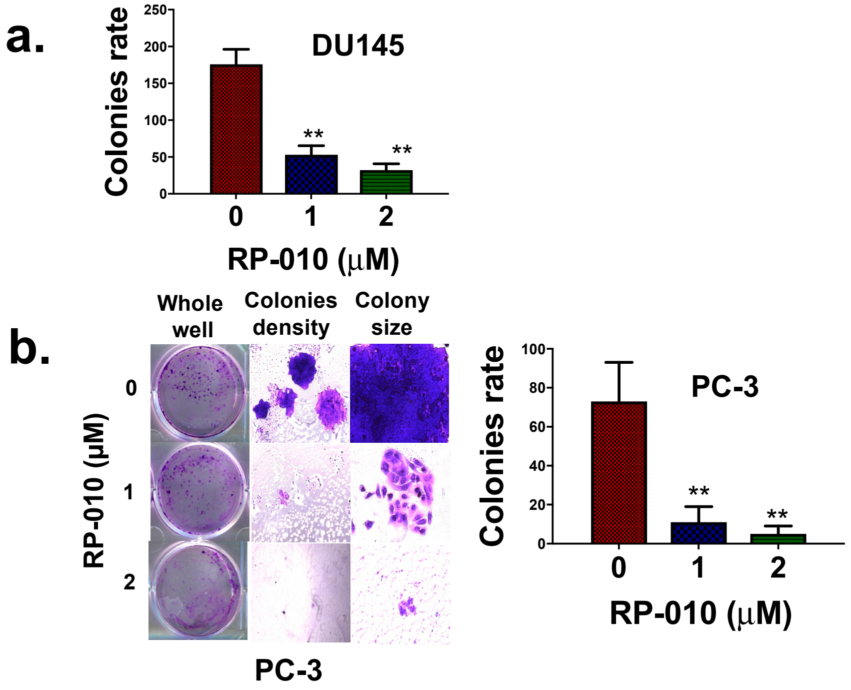

Figure S1. The effect of RP-010 on colony formation rate of prostate cancer cells. (**a**) A histogram showing the effect of RP-010 (1 or 2 μM) and vehicle on the number of colonies formed in DU145 cells. **(b)** The effect of RP-010 (1, or 2 μM) and vehicle on colony density (10×) and colony size (20×) of PC-3 cells and a histogram quantitating the results. All results are presented as the means ± SD of three independent experiments. ** $p < 0.01$.

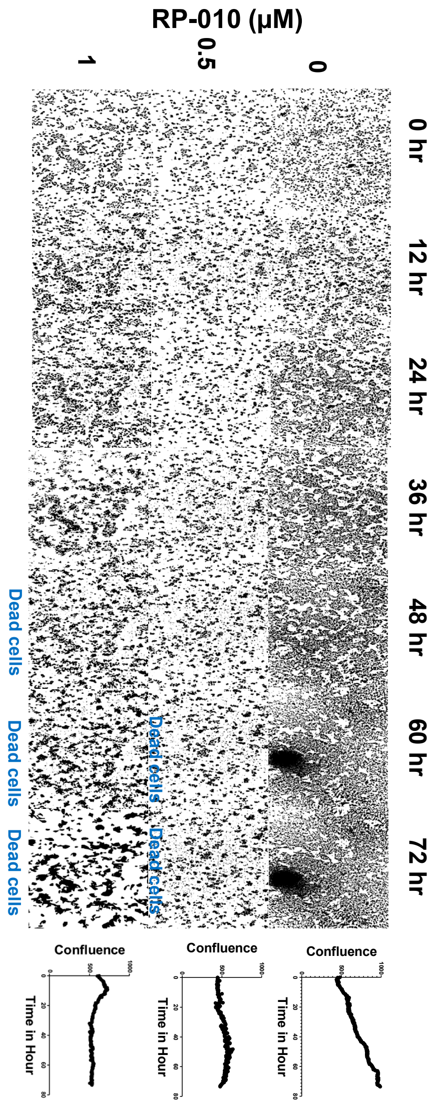

Figure S2. The effect of RP-010 on cell confluence and viability overtime in DU145 cells. DU145 cells were incubated with RP-010 (0.5 or 1 μM) or vehicle for 72 hrs. The images represent cell confluence over time. The graphs adjacent to the pictures represent the quantification of cell confluence over time. Dead cells refer to detached cells that were clearly floating.

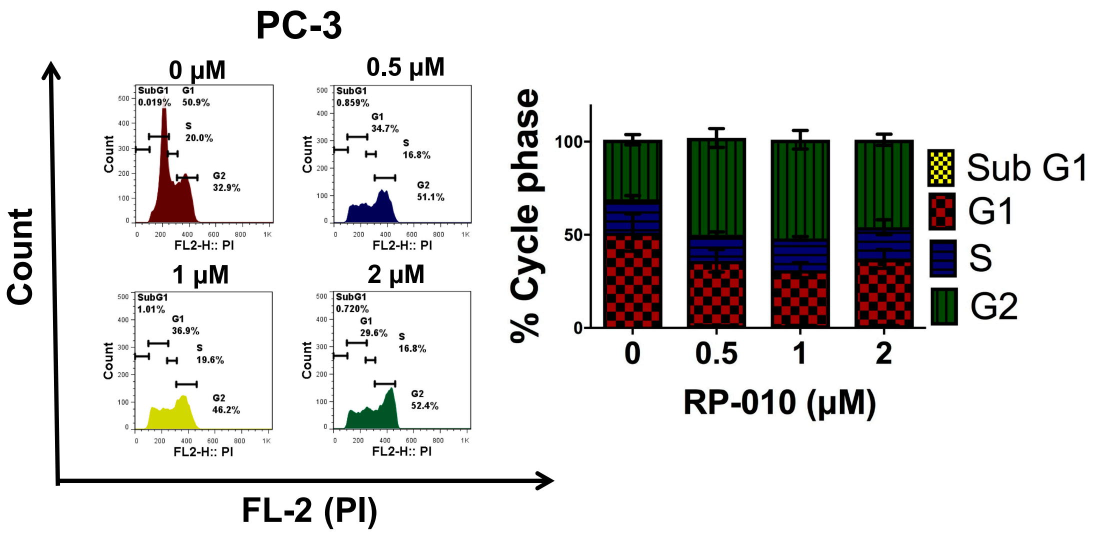

Figure S3. RP-010-induced alterations in the cell cycle in PC-3 cells. The analysis of RP-010 (0.5, 1, and 2μM) -induced alterations in the cell cycle using flow cytometric assay (P1 on Y axis and cell count on x axis). A graph showing the percent change for each phase of the cell cycle for RP-010.

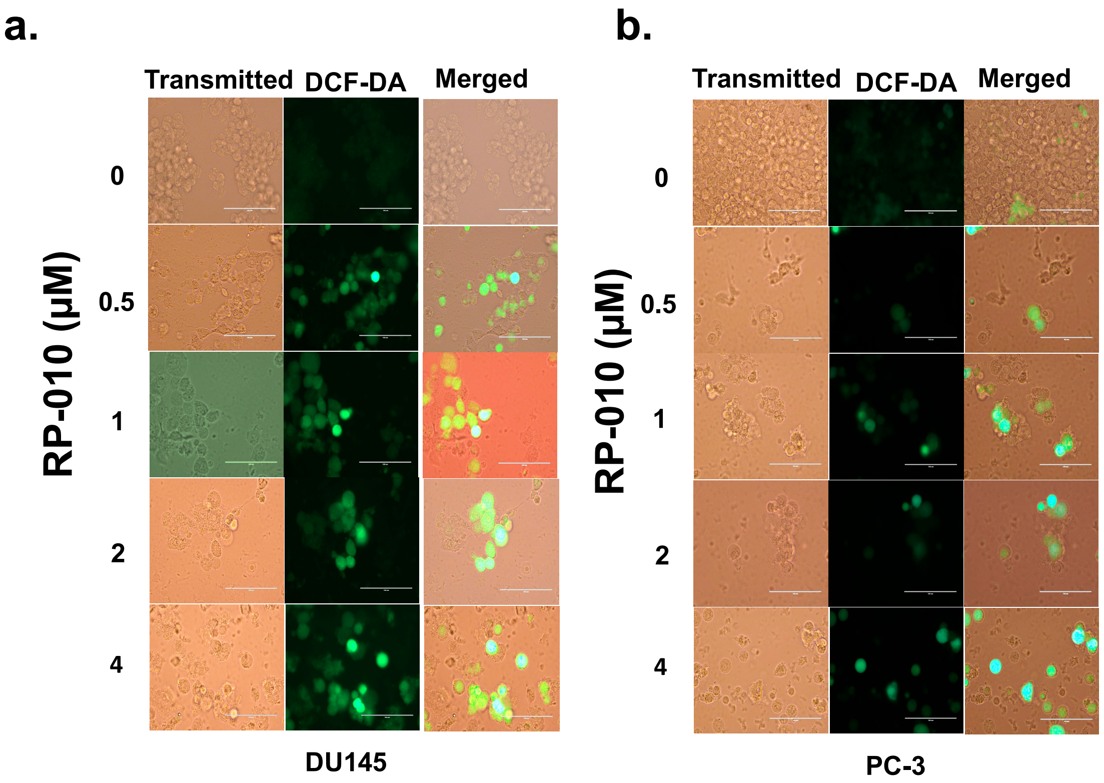

Figure S4. The effect of RP-010 on oxidative stress in DU145 and PC-3 cells. (**a**,**b**) Representative images of the DCF fluorescence after incubation with 0.5, 1, 2 and 4 μM of RP-010 or vehicle (0 uM) for 24 h in DU145 and PC-3 cells, respectively. The results are relative to the control. The experiments were repeated in triplicate. Scale bar: Images were taken at 4× (scale bar: 1000 μm).

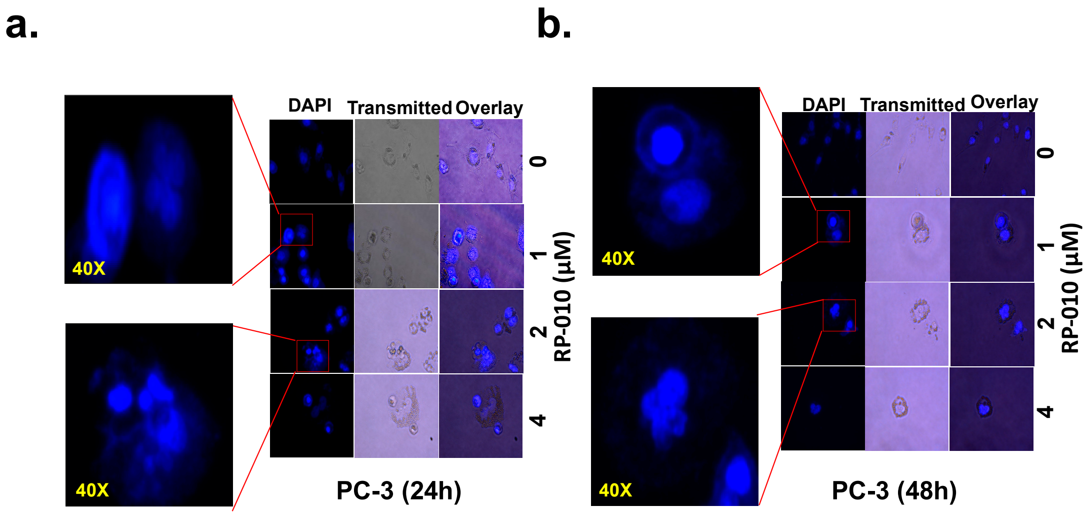

Figure S5. The effect of RP-010 on nuclear events in PC-3 cells. The effect of RP-010 (1, 2 or 4 µM) or vehicle on the nucleus of PC-3 cells after incubation for (**a**) 24 or (**b**) 48 h, respectively. Both chromatin condensation and mitotic catastrophe can be seen.

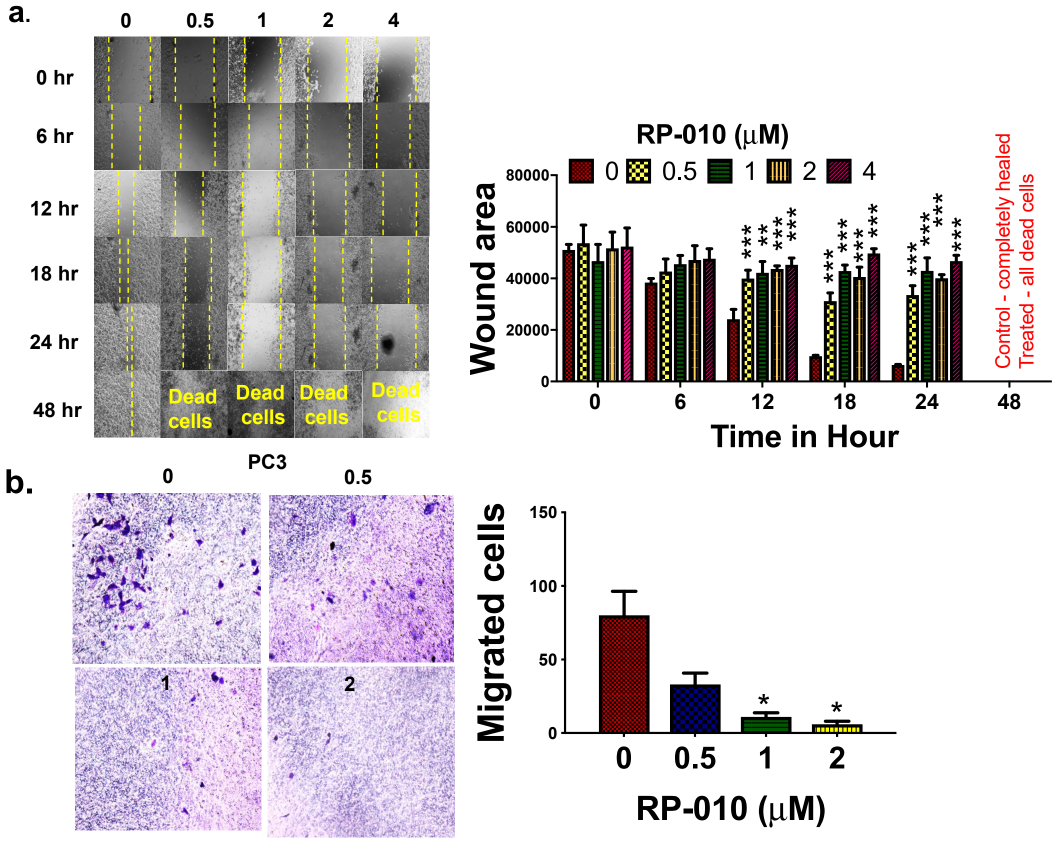

Figure S6. RP-010 significantly reduces cell migration in PC-3 cells (**a**) The pictures show the results of the wound healing assay after incubation with RP-010 (0.5, 1, 2, or 4µM) or vehicle. The results are also summarized in the adjacent histogram (**b**) the pictures show the transwell migration of

cells after incubation with RP-010 (0.5, 1 or 2 μM) or vehicle. The results are summarized in the adjacent histogram.

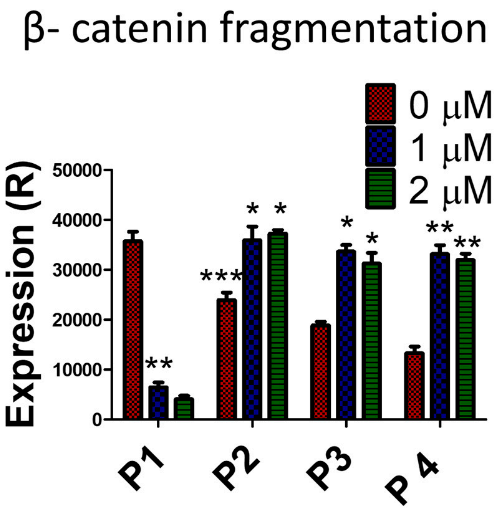

Figure S7. RP-010 induces significant changes in Wnt/β-catenin signaling. The effect of RP-010 (1 or 2 μM) or vehicle on β-catenin fragmentation. (R) = relative.

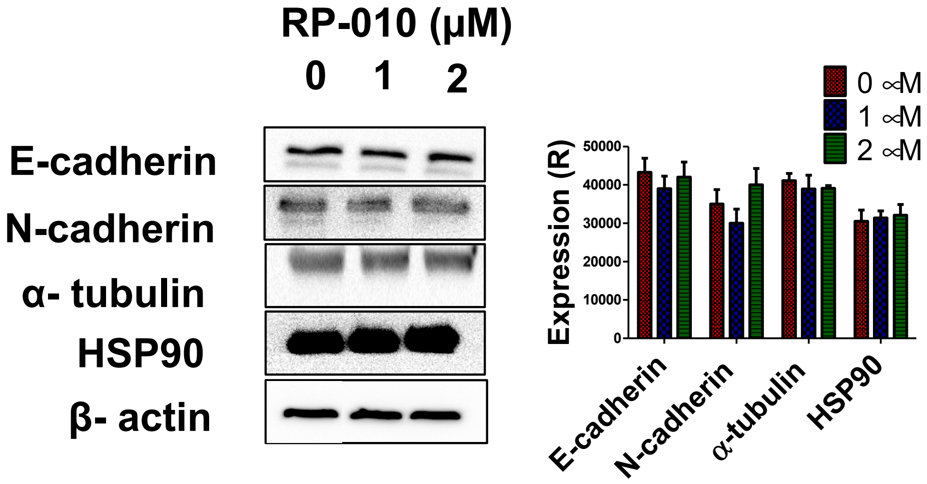

Figure S8. The effect of RP-010 (1 or 2 μM) or vehicle on the expression of E-cadherin, N-cadherin, α-tubulin, and HSP90. RP-010 was added at 0, 1, 2 μM concentrations. β-actin was used as a reference protein. (R) = relative.

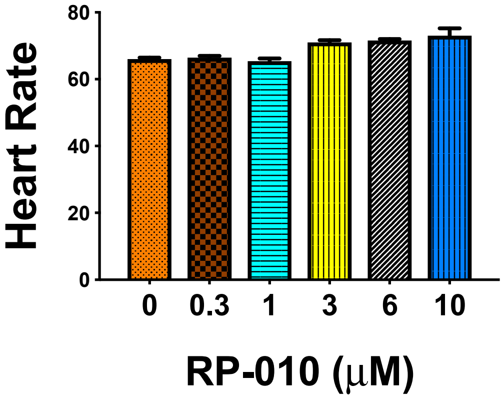

Figure S9. The effect on the heart rate of zebra fish exposed for upto 48 h with (0.3, 1, 3, 6 or 10 μM) or without RP-010.

© 2019 by the authors. Licensee MDPI, Basel, Switzerland. This article is an open access article distributed under the terms and conditions of the Creative Commons Attribution (CC BY) license (http://creativecommons.org/licenses/by/4.0/).
